# Histopathology of the Liver, Kidney, and Spleen of Mice Exposed to Gold Nanoparticles

**DOI:** 10.3390/molecules23081848

**Published:** 2018-07-25

**Authors:** Khalid Elfaki Ibrahim, Mohsen Ghaleb Al-Mutary, Amel Omer Bakhiet, Haseeb Ahmad Khan

**Affiliations:** 1 Department of Zoology, College of Science, King Saud University, Riyadh 11451, Saudi Arabia; kibrahim@ksu.edu.sa; 2 Department of Basic Sciences, College of Education, Imam Abdulrahman Bin Faisal University, Dammam 34212, Saudi Arabia; mgalmutary@iau.edu.sa; 3 Deanship of Scientific Research, Sudan University of Science and Technology, Khartoum 11111, Sudan; amel33@gmail.com; 4 Department of Biochemistry, College of Science, King Saud University, Riyadh 11451, Saudi Arabia

**Keywords:** gold nanoparticles, histopathology, liver, kidney, spleen, mice, priming dose

## Abstract

Gold nanoparticles (GNPs) are biocompatible nanomaterials that are currently researched for biomedical applications such as imaging and targeted drug delivery. In this investigation, we studied the effects of a single dose (injected on day 1) as well as a priming dose (two injections with a gap of one week) of 5 nm, 20 nm, and 50 nm diameter GNPs on the structural and biochemical changes in the liver, kidney, and spleen of mice. The results showed that small sized GNPs (5 nm) produced significant pathological changes in the liver on day 2 that gradually reduced on day 8. The medium (20 nm) and large (50 nm) sized GNPs preferentially targeted the spleen and caused significant pathological changes to the spleen architecture on day 2 that persisted on day 8 as well. There were minimal and insignificant pathological changes to the kidneys irrespective of the GNPs size. The animals that were primed with the pre-exposure of GNPs did not show any aggravation of histological changes after the second dose of the same GNPs. None of the dose regimens of the GNPs were able to significantly affect the markers of oxidative stress including glutathione (GSH) and malondialdehyde (MDA) in all of the organs that were studied. In conclusion, the size of GNPs plays an important role in their pathological effects on different organs of mice. Moreover, the primed animals become refractory to further pathological changes after the second dose of GNPs, suggesting the importance of a priming dose in medical applications of GNPs.

## 1. Introduction

After the advent of nanotechnology, there is a growing trend about the design, synthesis, and use of engineered nanoparticles (NPs) in different areas including medicine, cosmetics, coating, bioremediation, paints, electronics, and the food industry [1,2,3,4,5,6,7,8]. Recently, gold nanoparticles (GNPs) have been regarded as promising candidates for optical sensors, imaging, drug delivery, and therapeutic applications due to their size- and shape-dependent physical properties and their inherent biocompatibility compared with other metallic nanoparticles [9,10,11,12].

The efficient free radical scavenging activity of resonantly illuminated GNPs significantly decreased dialysis-induced oxidative stress, as well as reduced some acute adverse effects that were caused by dialysis-induced protein adsorption, platelet adhesion, and activation of coagulation, thus avoiding thrombosis during hemodialysis, which demonstrates their translational potential for chronic renal disease [13]. In a murine model of asthma, the instillation of GNPs prevented airway hyper-reactivity, inflammation, and lung remodeling by reducing the lung tissue generation of pro-inflammatory cytokines and chemokines with a mechanism that was probably related to the down-regulation of oxidative stress [14]. Muller et al. [15] observed that GNPs not only prevented intracerebroventricular streptozotocin-induced impairment in mitochondrial ATP production, neuroinflammation, and oxidative stress, but also protected the rats against memory deficits, suggesting that GNPs may be considered as a potential treatment for dementia.

As the use of GNPs is gaining momentum for a variety of applications, their concentrations are likely to increase in the environment, posing an emerging threat to the environment and the food chain. Thorough characterization of the toxic effects of NPs is important due to the increasing risk of potential environmental contamination by NPs and its associated adverse effects [16]. Traditionally, gold (Au) is considered as a poorly reactive or chemically inert material and is anticipated as biocompatible in living organisms. However, the conversion of a bulk material into a nano scale imparts unique properties including a large surface area, high reactivity, and strong interaction with biological matrices, due to their small size. Hence, the question as to whether GNPs have potential toxicological effects on human health remains to be answered [17].

A comparative evaluation of the biodistribution and the toxic profiles of GNPs and silver NPs showed that their chemical composition played a critical role in their in-vivo biodistribution and toxicity [18]. Particularly, GNPs were prominently stored in the liver, whereas AgNPs preferentially accumulated more in organs such as the heart, lung, and kidneys. Capping of GNPs with a biopolymer (chitosan) modified the inherent positive charges and hydrophobicity, with the resultant boosting in the production of reactive oxygen species (ROS) and misbalances of the antioxidant parameters of parasites. The excessive generation of ROS induced oxidative stress, leading to apoptotic cell death in filarial worms with negligible toxicity to human PBMCs, suggesting the potential application of biopolymer-capped GNPs as efficient antifilarial therapeutics [19]. Baudrimont et al. [20] addressed the environmental impact of trophic transfer and the adverse effects of functionalized GNPs from periphytic biofilms to the crustacean Gammarus fossarum. The crustaceans were allowed to graze on biofilms, which were pre-exposed for 48 h to 10 nm positively charged functionalized GNPs (4.6 and 46 mg/L) for 7 days. The findings showed cellular damage caused by oxidative stress and, in particular, an adverse impact on mitochondrial respiration [20].

Gold nanoparticles of 50 nm diameter were found to be highly effective against MCF7 breast cancer cells due to their superior penetration in cultured cells and their effective accumulation in tumor xenografts after intravenous injection, whereas larger GNPs were mainly localized in the tumor periphery instead of penetrating deep into the tumors [21]. Gold nanospheres appeared to be nontoxic in human keratinocyte cells whereas gold nanorods caused significant generation of ROS and the upregulation of genes that are involved in cellular stress and toxicity, suggesting that shape also plays a key role in mediating the cellular response following exposure to GNPs [22]. Although the systemic toxicity of the intermediate sized citrate-capped GNPs was linked to major organ damage in mice, the same GNPs appeared to be nontoxic in vitro using HeLa cell lines [23]. Thus, it is of prime concern to investigate the in vivo effects of nanoparticles before approving their use for clinical applications [24].

In a previous study on mice, intraperitoneally injected GNPs (12.5 nm, 40–400 μg/kg/) that were administered daily for 8 consecutive days were largely taken up by the spleen, kidney, and liver [25]. There were no abnormal histopathological findings in the liver, kidney, and spleen after a 14-day repeated oral administration of GNPs or Au ions up to the dose of 1300 μg/kg [16]. The repeated exposure of 14 nm GNPs for 56 days followed by a 14 day washout period showed their bioaccumulation without causing any structural or functional impairment in the liver and kidneys of the rats [26]. Whereas, Abdelhalim and Jarrar [27] observed significant abnormalities in the liver pathology after repeated doses (3–7 days) of 10, 20, and 50 nm GNPs in rats. Chen et al. [23] have also demonstrated pathological abnormalities in the liver, spleen, and lungs of mice after intraperitoneal injections of GNPs (8–37 nm) at a dose of 8 mg/kg/week.

The above literature indicates that most of the previous studies have focused on studying the effects of repeated exposure of GNPs, whereas the acute toxicity of a single dose of GNPs of different sizes has not been tested. We therefore investigated the effects of a single dose as well as a priming dose (two injections with a gap of 6 days) of GNPs on the structural changes in the liver, kidney, and spleen of mice. Considering the fact that drug-laden GNPs subsequently become remnant after the drug release in the body, we used naked GNPs for this study. We chose three different sizes of GNPs (5 nm, 20 nm, and 50 nm diameter). Although 20–50 nm GNPs are considered appropriate as drug carriers, small sized GNPs (5 nm) may have greater potential for imaging. We also performed biochemical analysis of markers of oxidative stress in the liver, kidney, and spleen of mice that were exposed to GNPs.

## 2. Materials and Methods

### 2.1. Animals and Treatment Groups

Adult Swiss albino female mice, aged 7 ± 0.5 week and weighing 30 ± 5 g, were housed in a temperature- and humidity-controlled facility and were maintained on a 12 h light/dark cycle. Standard laboratory mice chow diet and tap water were freely available ad libitum to the animals. The mice were randomly divided into 10 groups with 6 animals in each group. One of the groups served as the control and received the vehicle only. The animals in 3 of the treatment groups were treated with a single intraperitoneal injection of 5 nm, 20 nm, and 50 nm GNPs, respectively, and were sacrificed on the next day. Another 3 groups received the same treatment of GNPs as mentioned above, however these animals were sacrificed after 7 days. The last 3 groups served as priming dose groups and received the same treatment as mentioned above, however in addition, they also received a second injection of GNPs on day 7 and were sacrificed on day 8. The experimental protocol was approved by the Institutional Ethics Board (Ref. IEB/DSR/016, Dated 15/02/2017) and was conducted in accordance with the National Institute of Health guide for the care and use of laboratory animals while making efforts to minimize animal suffering.

### 2.2. Gold Nanoparticles

**Figure 1:**
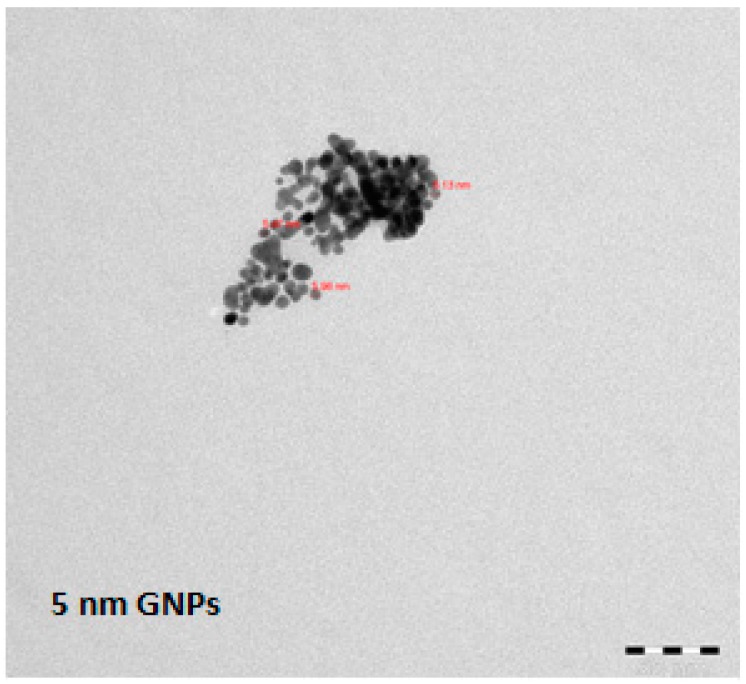

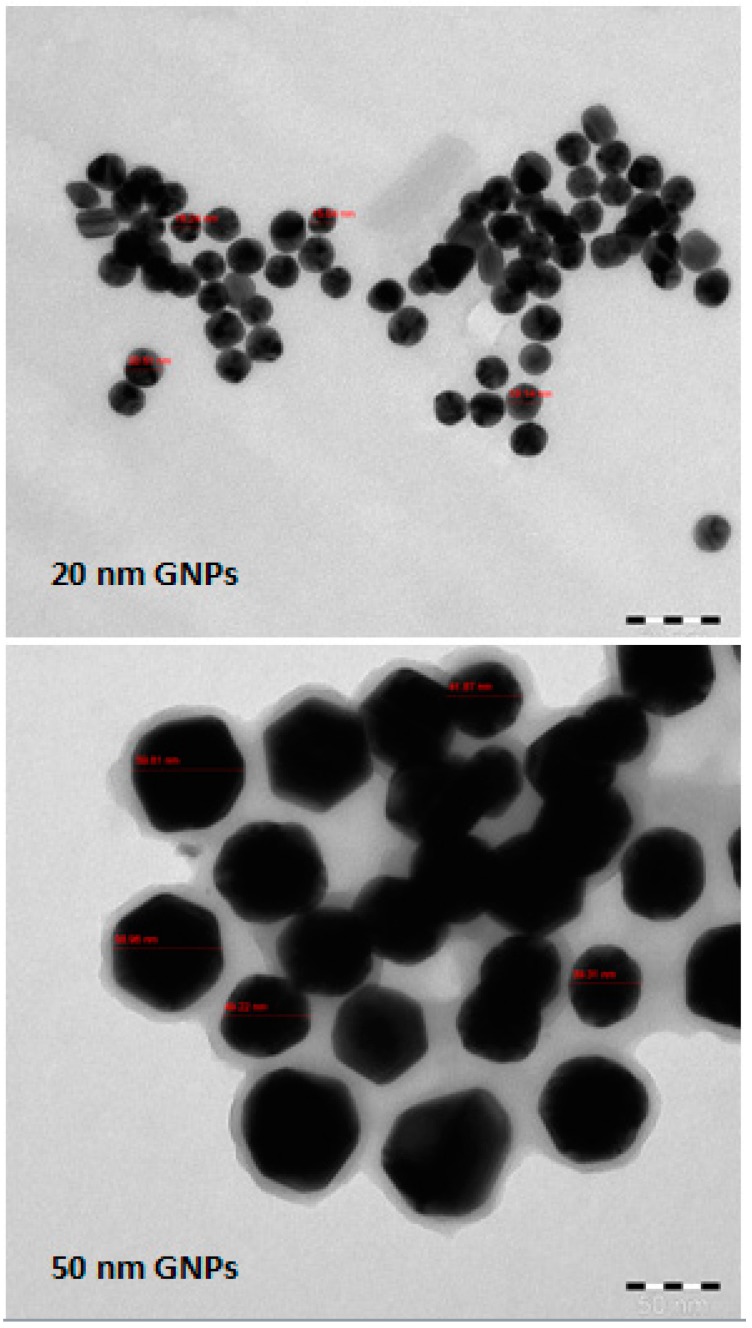
Evaluation of the size and shape of the GNPs using transmission electron microscopy. Scale bar = 50 nm.

Gold nanoparticles of 5 nm (Cat. No. MKN-Au-005), 20 nm (MKN-Au-020), and 50 nm (MKN-Au-050) diameter (Au concentration of 0.01%) which were stabilized with chloroauric acid (HAuCl_4_) were purchased from MK Impex Corporation, Missisauga, ON, Canada. The stock solutions of GNPs were kept refrigerated. The size and morphology of the GNPs were evaluated by transmission electron microscopy (TEM) (Model 1011CX, JEOL, Tokyo, Japan) and are shown in Figure 1. The small and medium sized GNPs (5 and 20 nm) had a round shape, whereas the shape of the larger sized GNPs (50 nm) appear as hexagonal (Figure 1).

### 2.3. Animal Dosing

The control animals received a single injection of 100 µL of normal saline intraperitoneally. Stock solutions of different sized GNPs (5, 20, and 50 nm) were sonicated to dissipate any aggregation of GNPs before their dilution with normal saline. GNPs were administered in the dose of 5 µg Au/100 µL/animal (approximately equivalent to 170 µg/kg bodyweight). A clear presentation of the different treatment groups, their dosing, and their sacrifice times is given in Table 1.

The animals were subjected to cardiac perfusion with saline under ether anesthesia. The specimens of the liver, kidney, and spleen were isolated and were immediately stored at −80 °C for biochemical analysis. Appropriately sized portions of the liver, kidney, and spleen were fixed in 10% neutral buffered formalin for histopathological analysis.

### 2.4. Histopathology

The fixed specimens of the liver, kidney, and spleen were processed overnight for dehydration, clearing, and impregnation using an automatic tissue processor (Sakura, Japan). The specimens were embedded in paraffin blocks using an embedding station (Sakura, Japan) and serial sections of 4 µm thickness were cut using a microtome (ModelRM2245, Leica Biosystems, Wetzlar, Germany). We used an autostainer (Model 5020, Leica Biosystems, Wetzlar, Germany) for Hematoxylin & Eosin staining of the sections. The mounted specimens were observed and were scored under light microscopy. For a semi-quantitative comparison of the structural changes, the abnormalities in the tissue sections were graded from 0 (normal structure) to 3 (severe pathological changes).

### 2.5. Analysis of Glutathione (GSH)

The measurement of reduced glutathione (GSH) in the different organ tissues (liver, kidney, spleen) was performed according to the procedure that was reported earlier [28]. The tissue was homogenized in ice-cold perchloric acid (0.2 M) containing 0.01% of ethylenediamine tetraacetic acid (EDTA). The homogenate was centrifuged at 9000× *g* for 5 min in a refrigerated centrifuge (4 °C). The enzymatic reaction was started by adding 100 µL of clear supernatant into a spectrophotometric cuvette containing 800 µL of 0.3 mM reduced nicotinamide adenine dinucleotide phosphate (NADPH), 100 µL of 6 mM 5,5-dithiobis-2-nitrobenzoic acid (DTNB), and 10 µL of 50 units/mL glutathione reductase (all of these reagents were freshly prepared in a phosphate buffer at pH 7.5). The absorbance was measured over a period of 120 s at 412 nm at 30 °C. The GSH level was determined by comparing the rate of the change of absorbance of the test solution with that of the standard GSH.

### 2.6. Analysis of Malondialdehyde (MDA)

The level of MDA in the different organs of the rats was analyzed spectrophotometrically, as described earlier [28]. The tissues were weighed and homogenized (10% *w*/*v*) in ice-cold 0.15 M potassium chloride in an ultraturax homogenizer. Tissue homogenate (1 mL) was incubated at 37 °C in a metabolic shaker for 2 h. One milliliter of 10% (*w*/*v*) trichloroacetic acid was mixed with homogenate, followed by centrifugation at 3000 rpm for 10 min. Aliquots (1 mL) of the clear supernatant were mixed with 1 mL of 0.67% (*w*/*v*) 2-thiobarbituric acid and were placed in a boiling water bath for 10 min, and were then cooled and diluted with 1 mL distilled water. The absorbance of the solution was recorded at 535 nm, and the concentration of MDA was calculated using tetraethoxypropane as an external standard.

### 2.7. Statistics

The data were analyzed by a one-way analysis of variance (ANOVA) followed by Dunnett’s multiple comparison test using the SPSS statistical package. *p* values less than 0.05 were considered as statistically significant.

## 3. Results

**Figure 2:**
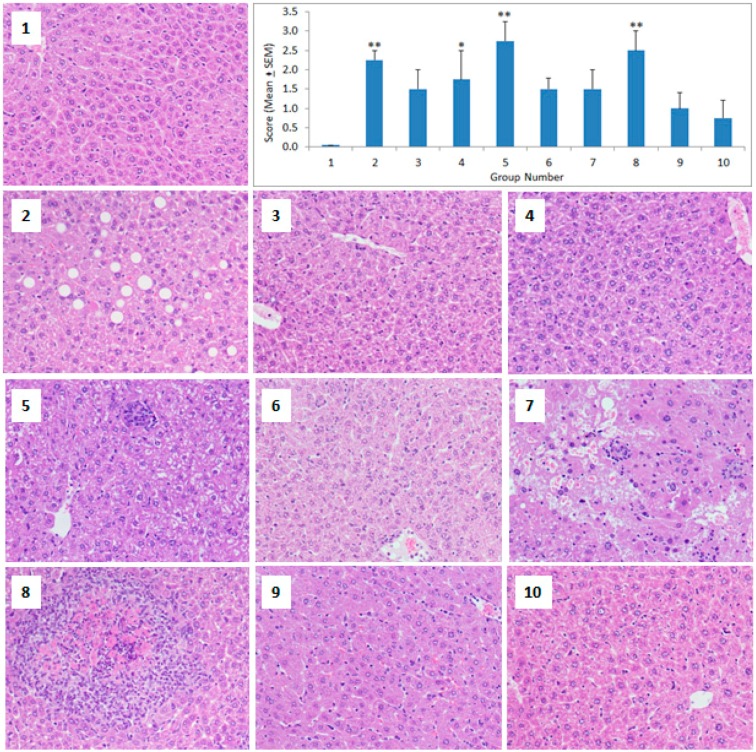
Histopathology of the liver. Light micrographs of the liver sections from different treatment groups. The numbers on the images represent the treatment groups according to Table 1. (**1**) Control mouse liver section showing normal hepatic architecture, (**2**) marked pathological changes characterized by steatosis and the abundance of micro and macro vesicles, (**3**) and (**4**) look healthy with normal hepatocytes while some bi-nucleated cells refer to regeneration, (**5**) cytoplasmic degeneration and some aggregation of inflammatory cells, (**6**) looks healthy with mild activation of Kupffer cells, (**7**) multi necrotic foci filled with hemorrhage and also the presence of infiltrative cells, (**8**) necrotic foci filled with edema and surrounded by inflammatory cells, (**9**) and (**10**) look healthy with bi-nucleated cells and the activation of Kupffer cells. The bar graph shows the scoring of the pathological changes. * *p* < 0.05 and ** *p* < 0.01 versus the control group using Dunnett’s multiple comparison test. (Magnification 200×).

A single injection of 5 nm GNPs caused significant pathological changes in the mice liver on day 2 that persisted on day 8 (ANOVA *F* = 4.124, *p* = 0.002) (Figure 2). The GNPs of 20 nm and 50 nm diameter produced comparatively less pathological changes in the liver as compared to the 5 nm GNPs. The second injection (priming groups) of the GNPs did not increase the severity of the pathological changes in the livers of the respective groups (Figure 2). The histopathological changes in the livers of the GNP-treated mice appeared in the form of steatosis, micro and macro vesicles, cytoplasmic degeneration, necrotic foci, Kupffer cells activation, hemorrhage, and infiltration of inflammatory cells. In some cases, bi-nucleated cells were observed that indicated the process of regeneration (Figure 2).

**Figure 3:**
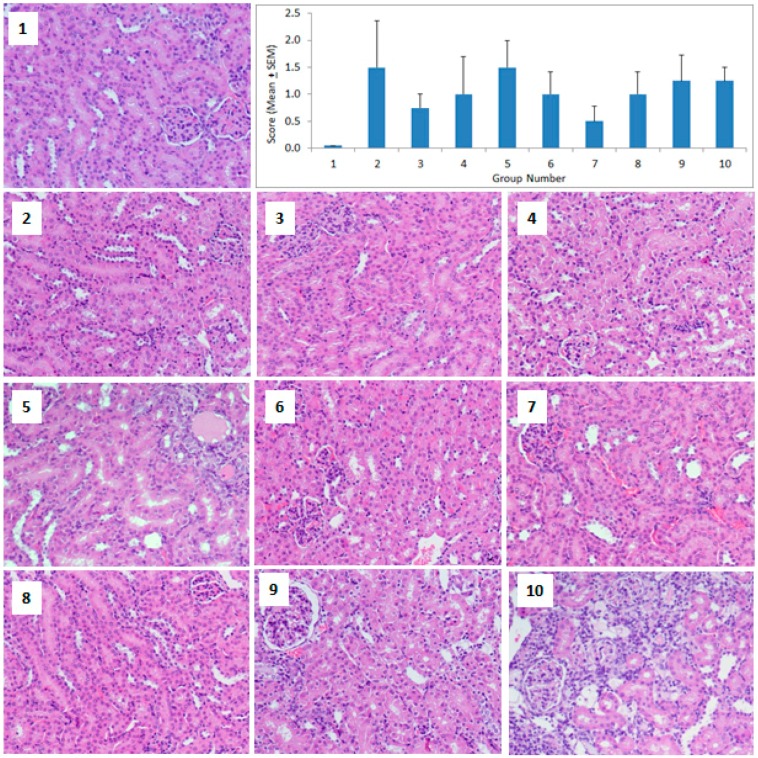
Histopathology of the kidney. Light micrographs of the kidney sections from the different treatment groups. The numbers on the images represent the treatment groups according to Table 1. (**1**) Control kidney section showing normal renal cortex and glomerular tufts, (**2**), (**3**), and (**4**) diminished and distorted glomeruli, (**5**) leukocyte infiltration, edema exudate, and necrotic foci, (**6**) distorted glomeruli, (**7**) relatively healthy glomeruli and tubules, (**8**) diminished and distorted glomeruli and dilated tubules, (**9**) relatively healthy glomerulus with abundant capsular space, (**10**) infiltration of inflammatory cells surrounding the distorted glomeruli and tubules. The bar graph shows the scoring of the pathological changes. (Magnification 200×).

Kidney histopathology showed that the exposure of GNPs caused pathological changes in the form of diminished and distorted glomeruli, dilated tubules, edema exudate, mild necrosis, and infiltration of inflammatory cells (Figure 3). However, the histopathological changes in the kidneys did not reach statistical significance due to the high intragroup variations (ANOVA *F* = 0.945, *p* = 0.502).

**Figure 4:**
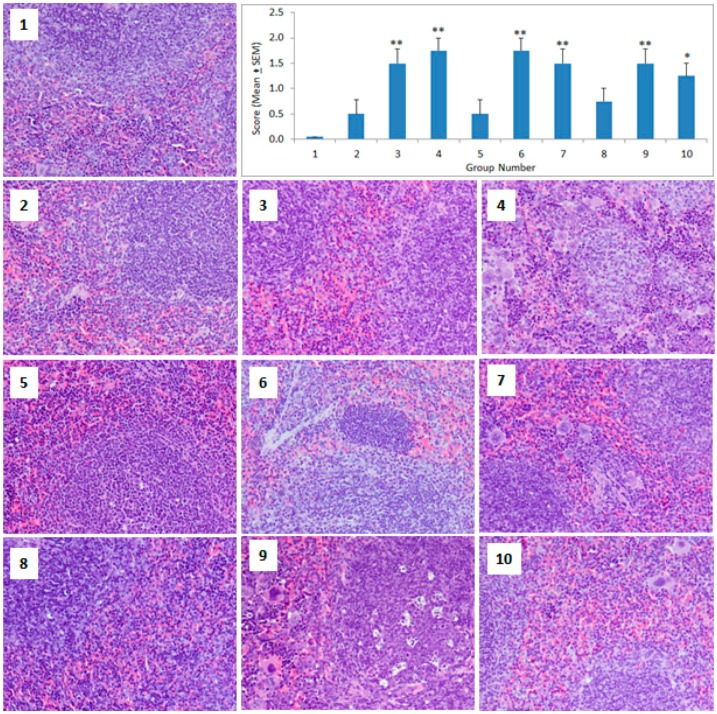
Histopathology of the spleen. Light micrographs of the spleen sections from the different treatment groups. The numbers on the images represent the treatment groups according to Table 1. (**1**) Control spleen section showing normal splenic architecture with normal lymphoid follicles and sinuses, (**2**) well-defined spleen section, (**3**) minimized lymphoid follicles, but still the white pulp is more well-defined than the red pulp, (**4**) ill-defined spleen section with diffuse white pulp, distorted lymphoid architecture, and giant macrophages, (**5**) well-defined spleen section with a healthy lymphoid follicle, (**6**) ill-defined spleen section, (**7**) lymphoid follicles surrounded by giant macrophages, (**8**) well-defined spleen section, (**9**) presence of granular leukocytes in between lymphocytes in lymphoid follicles besides giant macrophages, (**10**) presence of giant macrophages. The bar graph shows the scoring of the pathological changes. * *p* < 0.05 and ** *p* < 0.01 versus the control group using Dunnett’s multiple comparison test. (Magnification 200×).

In the spleen, the smaller sized GNPs (5 nm) did not produce any significant change in the cellular architecture, whereas the 20 nm and 50 nm GNPs caused significant pathological changes on day 2 that persisted on day 8 (ANOVA *F* = 5.667, *p* = 0.001) (Figure 4). The second dose of GNPs did not produce any additional damage in the spleen of the primed animals. The pathological changes in the spleen were observed in the form of distorted lymphoid architecture, minimized lymphoid follicles, diffuse white pulp, the presence of granular leukocytes, and giant macrophages (Figure 4).

The results of the biomarkers of oxidative stress including GSH and MDA are given in Table 2 and Table 3, respectively. None of the dose regimens of the GNPs were able to significantly affect the GSH levels in the liver (ANOVA *F* = 0.310, *p* = 0.968), kidney (ANVOA *F* = 0.671, *p* = 0.731) and the spleen (ANOVA *F* = 1.261, *p* = 0.282). There were no significant changes in the levels of MDA in the liver (ANVOA *F* = 1.137, *p* = 0.356), the kidney (ANVOA *F* = 0.408, *p* = 0.925), and the spleen (ANOVA *F* = 1.100, *p* = 0.381) of the mice that were treated with GNPs (Table 3).

## 4. Discussion

The results of this study showed that small sized GNPs (5 nm) produced more pathological changes in the liver (Figure 2), whereas the medium (20 nm) and large sized (50 nm) GNPs mainly targeted the spleen (Figure 4). In the primed animals, the second dose of GNPs did not exacerbate the pathological changes in the liver (Figure 2), kidney (Figure 3), or the spleen (Figure 4), indicating the protective capacity building in these animals against the re-exposure of the same GNPs. A recent in-vitro study on human dermal fibroblasts (HDFs) showed that an acute exposure of GNPs induced more gene expression changes than its chronic counterpart and that the stress effects that were caused by acute exposure sustained, even after 20 weeks without any additional GNP exposure [9]. Thus, an acute burst of exposure to GNPs is more harmful to cells, and cells can adapt to protect themselves against long-term nanoparticle exposure. Another in-vitro study on primary rat hepatocytes showed that both citrate and protein corona coated GNPs exerted only an acute effect on the albumin synthesis of hepatocytes, with no chronic impact [29]. Thus, the acute phase toxicity of GNPs after the first dose followed by the recovery phase, even after the subsequent exposure of GNPs, is in agreement with previous reports that have shown the absence of toxicity in animals that were treated with multiple doses of GNPs [16,26].

The administration of GNPs did not affect the markers of oxidative stress, including GSH (Table 2) and MDA (Table 3). Ferreira et al. [30] observed a significant reduction in MDA levels in the rats’ brains after the acute administration of 10 nm or 30 nm GNPs (70 µg/kg), whereas the superoxide dismutase activity was increased after the acute (1 day) and long-term (28 days) exposure of GNPs in rats. The oral administration of GNPs (50–150 μg/kg) significantly reduced acetaminophen induced hepato-renal injury in rats and normalized the markers of oxidative stress as well as liver and renal function [31]. In a Schistosoma mansoni infected mouse model of renal damage, treatment with GNPs (250–1000 μg/kg) significantly reduced the extent of histological impairment and renal oxidative injury, measured by renal GSH and MDA [32]. The exposure of an environmentally relevant concentration of 20 nm citrate-stabilized GNPs to marine bivalve Ruditapes philippinarum did not provoke oxidative damage and therefore cannot be considered toxic to this model organism [33]. The administration of GNPs functionalized with Sambucus nigra L. extract increased the muscle and systemic GSH/GSSG ratio and decreased MDA levels in the diabetic rats as compared to the non-diabetic group [34]. Besides increasing the antioxidant defense, the same treatment also reduced metalloproteinases activity and inflammation in the liver tissue of the diabetic rats. The oral administration of biogenic GNPs not only reduced the histological injury in hepatic, renal, and pancreatic tissues, but also alleviated the hyperglycemic condition by increasing serum insulin and reducing the oxidative stress in the diabetic rats [35]. The reports mentioned above indicate that GNPs alleviate toxicant-induced oxidant injury by reducing the oxidative stress and improving the antioxidant defense. In our study, we used GNPs alone without any concomitant treatment of a toxicant, and we observed that the levels of oxidative stress markers in all of the treatment groups were comparable with the control group. These findings suggest that the dose regimen of GNPs that were used in this study does not affect the free radical indices in the different organs of mice.

Several studies have shown an acute phase induction on proinflammatory cytokines in the liver and kidneys of rats that were exposed to GNPs [36,37,38], whereas primed animals showed protection against GNP-induced acute immune activation [39]. If the proinflammatory cascade remains effective for a longer duration, it may lead to cellular injury via the excessive generation of ROS. The intraperitoneal injection of 10 nm and 30 nm GNP caused oxidative stress and altered the energy metabolism in the liver, heart, and kidneys of the rats [40]. Following the intraperitoneal injections, 10 nm diameter GNPs significantly increased liver MDA without altering GSH levels in the rat liver on day 3 and day 7 post-dosing [28]. The oral administration of GNPs (1–2 μM) for 14 consecutive days significantly increased the ROS and the depletion of the antioxidant enzyme status in the erythrocytes and tissues of the mice, causing hepatic and renal toxicity, which was evident from liver and kidney function tests [41]. Oral dosing of 10 nm diameter GNPs for 2 weeks significantly decreased the catalase (CAT) and glutathione peroxidase (GPX) enzymes activities in the mice [42]. A single intraperitoneal injection of silica-coated GNPs (1100 µg/kg, 100 nm) increased the MDA level and decreased superoxide dismutase, catalase, and glutathione peroxidase activities in the rat lungs. These effects were exacerbated by the exposure of the static magnetic field and were also confirmed by histopathological study of the tissue damage [43].

Although GNPs have antioxidant and anti-inflammatory effects, they may exert toxicity in certain formulation or doses. Therefore, it is critical to study both the therapeutic and the toxic properties of GNPs. In general, naked GNPs are considered inert and often nontoxic, however they can become toxic after being coated with certain charged molecules, such as chitosan [44]. Notably, these effects are dependent on the core material of the particle, the cell type used for testing, and the growth characteristics of these cell culture model systems. In mice, the induction of DNA damage by citrate-bound 30 nm and 40 nm GNPs was size and dose dependent, whereas the surface functionalization with PVP reduced the toxic effects of GNPs [45]. The GNPs (20 nm diameter) that were administered every 48 h showed potential therapeutic benefits without toxicity, whereas the 24 h group showed marked parenchyma changes with cell necrosis and leukocyte infiltration [46]. After a single intravenous injection in the rats, the citrate- and pentapeptide-coated GNPs (20 nm, 700 μg/kg) were rapidly removed from the blood stream and accumulated mainly in the liver [47]. After oral administration in the rats, the GNPs slowly entered the blood stream, showing peak concentration at 10 h, whereas Au ions were rapidly (peak concentration at 1 h) and largely absorbed into the systemic circulation as compared with GNPs. The absorption of GNPs via the oral route was minimal (1.85%) and after 14 days of repeated oral administration of GNPs to the rats, the Au levels significantly increased in the kidneys, but did not in the liver, lung, or spleen [17].

## 5. Conclusions

The size of GNPs plays an important role in their pathological effects on different organs of mice. The small sized GNPs (5 nm) preferentially targeted the liver, whereas the medium (20 nm) and large (50 nm) sized GNPs significantly affected the spleen. The pathological changes in the liver appeared one day post-dosing of the GNPs, followed by the gradual regeneration of the normal tissue after one week. However, GNP-induced pathological changes in the spleen persisted on day 8 as well. There were minimal and insignificant pathological changes in the kidneys irrespective of the GNPs size. The animals that were primed with the pre-exposure of GNPs did not show any aggravation of histological changes after the second dose of the same GNPs, indicating the importance of a priming dose in medical applications of GNPs.

## Figures and Tables

**Table 1:** Experimental groups used in this study.

Group No.	Group Name	Dosage	Number of Injections	Day of Injections	Day of Sacrifice
		(5 µg Au/Animal)			
1	Control	100 µL saline	1	1	2
2	GNP 5 (day 1)	100 µL GNP 5 nm	1	1	2
3	GNP 20 (day 1)	100 µL GNP 20 nm	1	1	2
4	GNP 50 (day 1)	100 µL GNP 50 nm	1	1	2
5	GNP 5 (day 7)	100 µL GNP 5 nm	1	1	8
6	GNP 20 (day 7)	100 µL GNP 20 nm	1	1	8
7	GNP 50 (day 7)	100 µL GNP 50 nm	1	1	8
8	GNP 5 (day 1,7)	100 µL GNP 5 nm	2	1 and 7	8
9	GNP 20 (day 1,7)	100 µL GNP 20 nm	2	1 and 7	8
10	GNP 50 (day 1,7)	100 µL GNP 50 nm	2	1 and 7	8

**Table 2:** The effect of GNPs on GSH levels in different organs of mice.

Treatment Group	Liver	Kidney	Spleen
Control	4256.50 ± 482.58	1553.75 ± 245.16	3120.58 ± 381.88
GNP 5 (day 1)	4567.75 ± 368.64	1630.42 ± 181.99	2917.25 ± 100.46
GNP 20 (day 1)	4580.50 ± 263.99	1162.00 ± 210.81	2920.50 ± 185.36
GNP 50 (day 1)	4654.75 ± 269.26	1358.75 ± 147.53	2818.50 ± 076.92
GNP 5 (day 7)	4479.00 ± 481.80	1311.50 ± 163.33	3291.75 ± 188.92
GNP 20 (day 7)	4052.50 ± 226.11	1455.00 ± 357.16	3215.50 ± 150.63
GNP 50 (day 7)	4417.20 ± 089.86	1189.80 ± 103.53	2983.80 ± 179.55
GNP 5 (day 1,7)	4510.50 ± 418.60	1422.75 ± 138.21	3254.75 ± 267.70
GNP 20 (day 1,7)	4175.00 ± 394.74	1248.75 ± 105.21	3098.00 ± 176.51
GNP 50 (day 1,7)	4321.80 ± 269.27	1628.00 ± 261.17	3595.00 ± 163.97

GSH levels are in nmoles/g wet tissue and are presented as mean ± standard error.

**Table 3:** The effect of GNPs on MDA levels in different organs of mice.

Treatment Group	Liver	Kidney	Spleen
Control	5.11 ± 0.44	7.11 ± 0.60	6.44 ± 0.23
GNP 5 (day 1)	5.08 ± 0.71	7.53 ± 0.96	6.28 ± 0.34
GNP 20 (day 1)	5.74 ± 0.59	7.62 ± 0.78	6.02 ± 0.35
GNP 50 (day 1)	7.12 ± 0.83	8.26 ± 1.31	5.81 ± 0.29
GNP 5 (day 7)	6.49 ± 0.91	7.50 ± 1.39	5.65 ± 0.28
GNP 20 (day 7)	6.30 ± 0.47	7.10 ± 1.10	5.58 ± 0.36
GNP 50 (day 7)	5.16 ± 0.72	6.72 ± 1.62	6.59 ± 0.37
GNP 5 (day 1,7)	7.00 ±1.19	5.84 ± 0.93	6.08 ± 0.28
GNP 20 (day 1,7)	6.97 ± 0.58	6.11 ± 1.46	6.04 ± 0.35
GNP 50 (day 1,7)	6.68 ± 0.92	6.84 ± 1.04	6.59 ± 0.51

MDA levels are in nmoles/g wet tissue and are presented as mean ± standard error.
